# Real-world diagnostic accuracy of lipoarabinomannan in three non-sputum biospecimens for pulmonary tuberculosis disease

**DOI:** 10.1016/j.ebiom.2024.105353

**Published:** 2024-09-26

**Authors:** Paul K. Drain, Xin Niu, Adrienne E. Shapiro, Zanele P. Magcaba, Zinhle Ngcobo, M William Ngwane, Katherine K. Thomas, Ronit R. Dalmat, Jennifer F. Morton, Elvira Budiawan, Abraham Pinter, Jason Cantera, Caitlin Anderson, Rose Buchmann, Doug Wilson, Ben Grant, Prove-TB Study team

**Affiliations:** a Department of Global Health, University of Washington, Seattle, WA, USA; b Department of Medicine, Division of Allergy and Infectious Diseases, University of Washington, Seattle, WA, USA; c Department of Epidemiology, University of Washington, Seattle, WA, USA; d Vaccine and Infectious Diseases Division, Fred Hutchinson Cancer Center, Seattle, WA, USA; e Umkhuseli Research and Innovation Management, Pietermaritzburg, South Africa; f Global Health Labs, Bellevue, WA, USA; g New Jersey Medical School, Rutgers University, Newark, NJ, USA; h University of KwaZulu-Natal, Pietermaritzburg, South Africa

**Keywords:** Tuberculosis, Lipoarabinomannan, Non-sputum, Electrochemiluminescence immunoassay, Diagnostic accuracy, HIV

## Abstract

**Background:**

Development of a non-sputum test using readily-obtainable biospecimens remains a global priority for tuberculosis (TB) control. We quantified lipoarabinomannan (LAM) concentrations, a pathogen biomarker for *Mycobacterium tuberculosis*, in urine, plasma and serum for real-world diagnostic accuracy of pulmonary TB among people living with and without HIV.

**Methods:**

We conducted a prospective diagnostic study among adults with TB symptoms in South Africa. We measured LAM concentrations in time-matched urine, plasma and serum with an electrochemiluminescence immunoassay using two capture antibodies (FIND 28 and S4–20). From the completed cohort, we randomly selected 210 participants (2 cases: 1 control) based on sensitivity estimates, and we compared diagnostic accuracy of LAM measurements against the microbiological reference standard.

**Findings:**

Urine and blood specimens from 210 of 684 adults enrolled were tested for LAM. Among 138 TB-positive adults (41% female), median urine LAM was 137 pg/mL and 52 pg/mL by FIND 28 and S4–20, respectively. Average LAM concentrations were highest in HIV-positive participants with CD4+ T cells <200 cells/mm^3^. Urine LAM by S4–20 achieved diagnostic sensitivity of 62% (95% CI: 53%–70%) and specificity of 99% (95% CI: 96%–100%). Plasma and serum LAM by FIND 28 showed similar sensitivity (70%, 95% CI: 62%–78%) and comparable specificities (90%, 95% CI: 82%–97%; 94%, 95% CI: 88%–99%). Diagnostic sensitivity of urine LAM by S4–20 was higher among participants without HIV (41%, 95% CI: 24%–61%) compared to HIV-positive participants with CD4 ≥200 cells/mm^3^ (20%, 95% CI: 8%–39%).

**Interpretation:**

Detection of LAM was achievable in non-sputum specimens for pulmonary TB, but additional analyte concentration or signal amplification may be required to achieve diagnostic accuracy targets.

**Funding:**

10.13039/100000865Bill and Melinda Gates Foundation.

**Research in context**

**Evidence before this study**

Despite being entirely curable with early detection, drug resistance testing, and consistent treatment, Tuberculosis (TB) remains the top cause of death from a single infectious agent worldwide. Inadequate TB diagnostics are still the key barrier to reduce disease burden. Sputum, as the most commonly used specimen type for TB diagnosis, can be difficult to obtain particularly in young children and people living with HIV (PLHIV). Sputum collection also poses potential risks to healthcare workers and proves ineffective in diagnosing extrapulmonary TB. To address current gaps in global TB control, World Health Organization (WHO) has thus been calling for the development of accurate TB biomarker tests using non-sputum samples and allowing same-day treatment initiation.

Lipoarabinomannan (LAM) is a cell wall glycolipid of *Mycobacterium tuberculosis* (*Mtb*) that is excreted and detectable in the urine of people with TB disease. With a pooled sensitivity at 42% and specificity at 92%, the Determine™ TB LAM Ag test was endorsed by WHO as a point-of-care (POC) test to detect urine LAM for TB diagnosis, but only among PLHIV. Its limited clinical sensitivity, failing to meet the minimal criteria in WHO's Target Product Profile (sensitivity ≥65% and specificity ≥98% regardless of people's HIV and CD4 status), has slowed the adoption and uptake of the Determine™ TB LAM Ag by national programs. The Fujifilm SILVAMP TB LAM (FujiLAM) is the next-generation urine LAM POC test that recently demonstrated similar specificity and an improved sensitivity by 20% among PLHIV when compared with the Determine™ TB LAM. However, significant variations in diagnostic accuracy by FujiLAM lot need to be addressed before further clinical use of the assay.

Multiple efforts are underway to develop a new, more accurate LAM assay for diagnosing TB disease among both PLHIV and people without HIV. Newly identified antibodies have been characterized using laboratory-based electrochemiluminescence (ECL) immunoassay. We searched PubMed for articles published in any language from database inception to January 31, 2024, for studies or reports of LAM detected by ECL for the diagnosis of TB. We used the following search terms: “(“tuberculosis” OR “tb”) AND (“lipoarabinomannan” OR “lam”) AND (“electrochemiluminescence”)”. Our search identified five original studies that evaluated the diagnostic performance of LAM by ECL. Using A194-01 as the common detector antibody, one retrospective study of 75 biobanked urine samples reported a sensitivity of 78% (95% CI: 62%–88%) for FIND 28 and 93% (95% CI: 80%–97%) for S4–20. The specificity was 63% (95% CI: 46%–77%) and 97% (95% CI: 85%–100%) respectively. A following study testing the serum samples matched with the 75 biobanked urine samples found a sensitivity of 55% (95% CI: 40%–69%) for FIND 28 and 30% (95% CI: 18%–45%) for S4–20, with the same specificity at 100% (95% CI: 90%–100%). In a more recent exploratory study of 24 urine samples, Cantera and collogues showed that antibody pair S4–20/A194-01 may have slightly better diagnostic performance than FIND 28/A194-01 for urine LAM detection. Compared with studies using biobanked samples unrepresentative of the real-world TB severity/LAM distribution, two prospective studies have also assessed the diagnostic accuracy of LAM by ECL for TB diagnosis. Yet, both were smaller studies that were conducted only among HIV-negative populations, with one from South Africa/Peru and the other one from Vietnam.

**Added value of this study**

To the best of our knowledge, this prospective study uniquely quantified LAM concentrations in three time-matched non-sputum biospecimens from a real-world clinical cohort of both PLHIV and HIV-negative people, using an ECL immunoassay. Employing two leading capture antibodies (FIND 28 and S4–20), our study further compared their diagnostic performance of LAM detected in different non-sputum biospecimens for pulmonary TB disease across populations with different HIV/CD4 status. We found that LAM detection in easy-to-collect non-sputum biospecimens was achievable with promising diagnostic accuracy for pulmonary TB disease. LAM concentrations were generally higher among PLHIV with immunosuppression, as compared to PLHIV with CD4 ≥200 cells/mm^3^ or participants without HIV. Detecting LAM by S4–20 in urine, or by FIND 28 in plasma/serum, nearly achieved diagnostic accuracy criteria to meet the WHO's TPP for rapid biomarker-based non-sputum tests. These findings provide valuable and generalizable insights into the diagnostic accuracy of LAM by ECL in real-world settings, and will help inform benchmark LAM targets for ongoing development of LAM-based rapid TB diagnostic tests.

**Implications of all the available evidence**

Regardless of people's HIV status or CD4 count, detection of LAM was feasible in easy-to-collect non-sputum biospecimens for pulmonary TB with promising diagnostic performance. However, to meet the diagnostic targets that not only measure accuracy but also consider population yields, prudent antibody selection by biospecimen type, and pre-analytical LAM concentration or signal amplification may be necessary.

## Introduction

Tuberculosis (TB) remains a leading cause of infectious mortality worldwide.^1^ This burden disproportionately affects persons living with HIV (PLHIV) in low- and middle-income countries (LMICs).^1^^,^^2^ With an annual gap of 4.2 million undiagnosed or unreported cases, inadequate diagnostics remain the major obstacle to mitigating the global burden of TB disease.^1^ Sputum-based molecular testing and culture are the gold standard for TB diagnosis, but obtaining lower respiratory specimens is resource intensive with poor diagnostic yield.^3^^,^^4^ In TB-endemic areas, less than 50% of hospitalized PLHIV can produce a sputum specimen, even with assistance.^5^, ^6^, ^7^ Thus, there is a global need for a rapid, accurate, non-sputum based diagnostic assay to accelerate TB diagnosis and treatment initiation.

Lipoarabinomannan (LAM) is a cell wall glycolipid of *Mycobacterium tuberculosis* (*Mtb*) that passes through the bloodstream before excretion in the urine of people with TB disease.^8^^,^^9^ The Determine™ TB LAM Ag assay for detection of urine LAM was approved by the World Health Organization (WHO) in 2015, and has proven to improve outcomes for immunosuppressed PLHIV.^10^, ^11^, ^12^ However, the Determine™ TB LAM Ag assay has not been approved for use among people without HIV, who comprise of the vast majority of global TB cases. Despite broadening the guidelines in 2019, the global adoption and uptake of the Determine™ TB LAM Ag has been limited by the assay's poor clinical sensitivity.^13^^,^^14^

Efforts are underway to develop a more accurate and sensitive second-generation LAM assay for diagnosing TB disease among PLHIV as well as people without HIV.^15^^,^^16^ Several newly identified LAM antibodies have been screened using an electrochemiluminescence (ECL) immunoassay, which allows for reliable quantification of low analyte concentrations in a small specimen volume.^17^, ^18^, ^19^ However, LAM concentrations in multiple concurrently-collected, non-sputum biospecimens for the diagnosis of pulmonary TB disease has not been described for both PLHIV and people without HIV from a real-world population. Thus, our objective was to compare the LAM concentrations across biospecimens and assess their diagnostic accuracy against a microbiological reference standard (MRS), when using several newly identified antibodies and a high-sensitivity ECL immunoassay. These results will help establish the potential for non-sputum LAM as a pathogen biomarker for pulmonary TB disease among both PLHIV and people without HIV, and inform benchmark LAM targets for ongoing development of LAM-based rapid TB diagnostic tests.

## Methods

### Study design and population

We conducted a prospective cohort study, called PROVE-TB, to evaluate the real-world LAM concentrations in time-matched urine, plasma, and serum biospecimens for diagnosis of pulmonary TB disease. We consecutively recruited adults with TB symptoms from the Harry Gwala Regional Hospital (formerly Edendale Hospital) inpatient wards and affiliated outpatient clinics in KwaZulu-Natal, South Africa between October 2019 and February 2021. Eligible persons were ≥16 years old and either had a recently documented HIV test result or agreed to test for HIV. We excluded persons who had received more than 24 h of TB treatment within the five days prior to study recruitment, and people who had competed TB treatment within the prior six months.

**Fig. 1:**
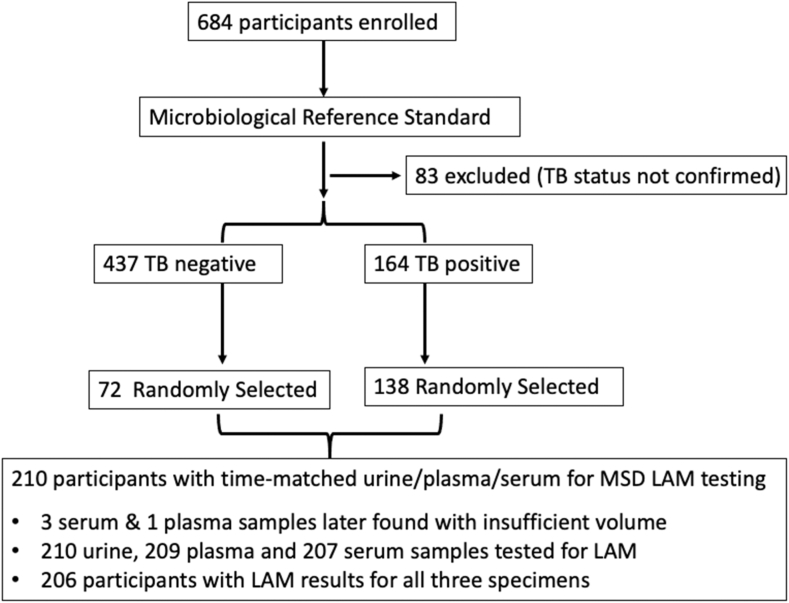
Participant flowchart and testing for LAM concentrations.

We enrolled 684 participants, of whom 83 were excluded for not having definitive sputum Xpert MTB/RIF Ultra (Cepheid, Sunnyvale, USA) and *Mtb* culture results (Fig. 1). Among the remaining 601 participants, we randomly selected participants in a 2:1 (case: control) ratio to obtain a selection of 210 participants for LAM testing.

### Ethics

All participants provided written informed consent. All laboratory testing was performed in a blinded fashion to minimize potential observer bias. No adverse events associated with specimen collection procedures were reported by participants. This study was approved by the ethical committees of the University of KwaZulu-Natal (BREC #BE475/18) and the University of Washington (Study 9092), and the study procedures complied with the ethical approvals.

### Microbiological reference standard for TB disease

Participants’ TB case status was determined by the Microbiological Reference Standard (MRS). A participant was considered to have met criteria of the MRS for TB disease (“TB-positive”) if at least one sputum specimen was positive by either molecular testing (Xpert MTB/RIF Ultra) or *Mtb* culture. To be classified as “TB-negative”, a participant was negative by sputum Xpert MTB/RIF Ultra, as well as by solid and liquid culture of any *Mycobacterium* spp. in sputa. The 83 participants who had incomplete MRS testing (i.e., either culture or Xpert result was missing and the other resulted negative) were considered “TB status not confirmed” and excluded from further LAM testing (Fig. 1).

### LAM testing by ECL

We measured LAM concentrations in triplicate for unconcentrated, time-matched urine, plasma, and serum specimens using an ECL immunoassay (MesoScale Diagnostics; Rockville, MD, USA). Immunoassays were conducted in a 2-assay U-PLEX SECTOR plate format (MesoScale Diagnostics).^18^^,^^19^ Using this format, as with lateral flow assays, requires one antibody for analyte capture and a second antibody for signal detection. We evaluated two of the best-performing LAM-capture antibodies, FIND 28 (Foundation for Innovative New Diagnostics, Geneva) and S4–20 (Otsuka Pharmaceuticals, Tokyo), based on our prior research of characterizing LAM antibodies. For all LAM immunoassays, we used the same detector antibody, A194-01 (Rutgers University, NJ, USA). All three antibodies have been studied and validated previously.^16^^,^^19^^,^^20^

To standardize ECL testing, we quantified the limit of detection (LOD) based on the standard-curve for each plate. The standard curve utilized LAM derived from irradiated H37Rv (BEI catalog NR-14848). An 11-point standard curve starting at 60 ng/mL with a threefold dilution factor for each subsequent dilution was used for urine samples. Plasma and serum standard curves were created similarly starting at 240 ng/mL with threefold dilution. Triplicates from the same sample that showed discrepancy (i.e., triplicates with LAM concentrations not consistently above or below the corresponding LOD), were tested two additional times. If a discrepancy persisted, the LAM concentrations were set to 0 pg/mL (i.e., similar to those with LAM results below the corresponding LOD from the same plate).

To account for possible LAM-negative samples having a signal higher than the blank buffer, we established another lab threshold, called the limit of blank (LOB). The LOB was set as the mean signal-to-background ratio (SBR) of five unblinded negative samples plus 1.645-fold the standard deviation of the SBR of the same five unblinded negative samples. LAM detected by each capture antibody was only considered a positive result if all LAM tests in triplicate were above the corresponding LOD and its mean SBR was greater than the LOB. Values for the LOB and the plate LODs are shown in Tables S1 and S2.

### Statistical analyses

We calculated descriptive statistics of demographic and clinical variables for participants with and without microbiologically-confirmed pulmonary TB disease. We summarized the LAM concentrations by specimen types and capture antibodies, as measured on the ECL platform. Density and violin plots were drawn to compare distributions of LAM concentrations (Log 10 transformed) in urine, plasma and serum. Since four participants had insufficient plasma or serum volume for LAM testing, all descriptive analyses were restricted to 206 participants with LAM results across all biospecimens. Our samples size calculation was based on achieving ± 8% sensitivity estimates for 95% confidence intervals, assuming diagnostic sensitivity was 65%.

We used Spearman's and Pearson's correlation coefficients to estimate the correlations between LAM results detected in different specimens. We also calculated standard diagnostic accuracy measures (sensitivity, specificity) for each biospecimen type and individual capture antibodies, as compared to the MRS definition for pulmonary TB disease. Stratified analyses for diagnostic accuracy were conducted based on HIV status and CD4+ T-cell counts (above vs below 200 cells/mm^3^). In addition to the lab detection thresholds (LOD & LOB), we performed Receiver Operating Characteristic (ROC) curve analyses and estimated the optimal cut off values that maximized the Area Under the Curve (AUC) to inform benchmark LAM targets.

To illustrate concordance between classifications of LAM detected by FIND 28 and S4–20, we plotted the Venn diagrams of the two capture antibodies for each specimen and by participant TB status. We also calculated the sensitivities and specificities of a potential parallel LAM test incorporating both capture antibodies for urine, plasma and serum respectively. Samples were considered as LAM positive if LAM was detected by either FIND 28 or S4–20 for each specimen type. Otherwise, samples were deemed as LAM negative in parallel testing. All statistical analyses were conducted in R (R Core Team, 2014).

### Role of the funding source

The study was funded by the Bill and Melinda Gates Foundation. The funder of the study (Bill and Melinda Gates Foundation) had no role in the data collection, data analyses, results interpretation, or writing of the report.

## Results

### Demographic and clinical characteristics

Among 684 participants enrolled, and after excluding 83 participants with incomplete MRS results, the cohort included 437 TB-negative and 164 TB-positive people (Fig. 1). Among those, 138 participants confirmed with TB disease and 72 participants without TB disease were randomly selected for LAM concentration testing. Among these 210 participants, the mean age was 40.2 years, 95 (45%) participants were female (self-reported), and 44% had HIV with CD4 <200 cells/mm^3^. Thirty-seven percent of participants reported a prior TB infection.

Among 138 with microbiologically-confirmed pulmonary TB disease, 109 (79%) were PLHIV and 67 (62%) had CD4 <200 cells/mm^3^ (Table 1). Among TB-positive participants, 46 (33%) tested urine LAM positive by the Determine TB LAM assay, whereas 3 (4%) TB-negative participants were positive by the assay. More TB-negative PLHIV had a CD4 ≥200 cells/mm^3^, as compared to TB-positive PLHIV (63% vs 27%).

**Table 1:** Participant characteristics at the time of specimen collection and TB testing (with matched urine/plasma/serum LAM results available, N = 206).

	Microbiologic reference standard	
	TB-positive N = 138	TB-negative N = 68
	N (%)	N (%)
**Demographic**		
Age, mean (±SD) in years	40 (±14)	41 (±12)
Female sex	57 (41%)	35 (51%)
**Clinical**		
Prior TB disease	51 (37%)	25 (37%)
Currently smoke tobacco	19 (14%)	13 (19%)
TB related symptoms		
Cough	116 (84%)	53 (78%)
Fever	98 (71%)	47 (69%)
Night sweats	97 (70%)	35 (51%)
Weight loss	123 (89%)	57 (84%)
Having any TB-related symptom	134 (97%)	65 (96%)
**Recruitment location**		
Inpatient ward	83 (60%)	68 (100%)
Outpatient clinic	55 (40%)	0 (0%)
**HIV status**		
HIV+	109 (79%)	68 (100%)
HIV+ with CD4 <200 cells/mm^3^	67 (62%)	23 (34%)
HIV+ with CD4 ≥200 cells/mm^3^	30 (27%)	43 (63%)
HIV+ with CD4 missing	12 (11%)	2 (3%)
**Tuberculosis testing**		
Urine determine TB LAM positive	46 (33%)	3 (4%)
Sputum smear microscopy positive^a^	9 (21%)	5 (31%)
**TB microbiological reference standard**		
Sputum Xpert ultra positive	131 (95%)	0 (0%)
Sputum *Mtb* culture positive	81 (59%)	0 (0%)

SD, standard deviation; TB, tuberculosis.

a Only 43 TB-positive and 16 TB-negative participants received sputum smear microscopy.

### Descriptive LAM concentrations

**Fig. 2:**
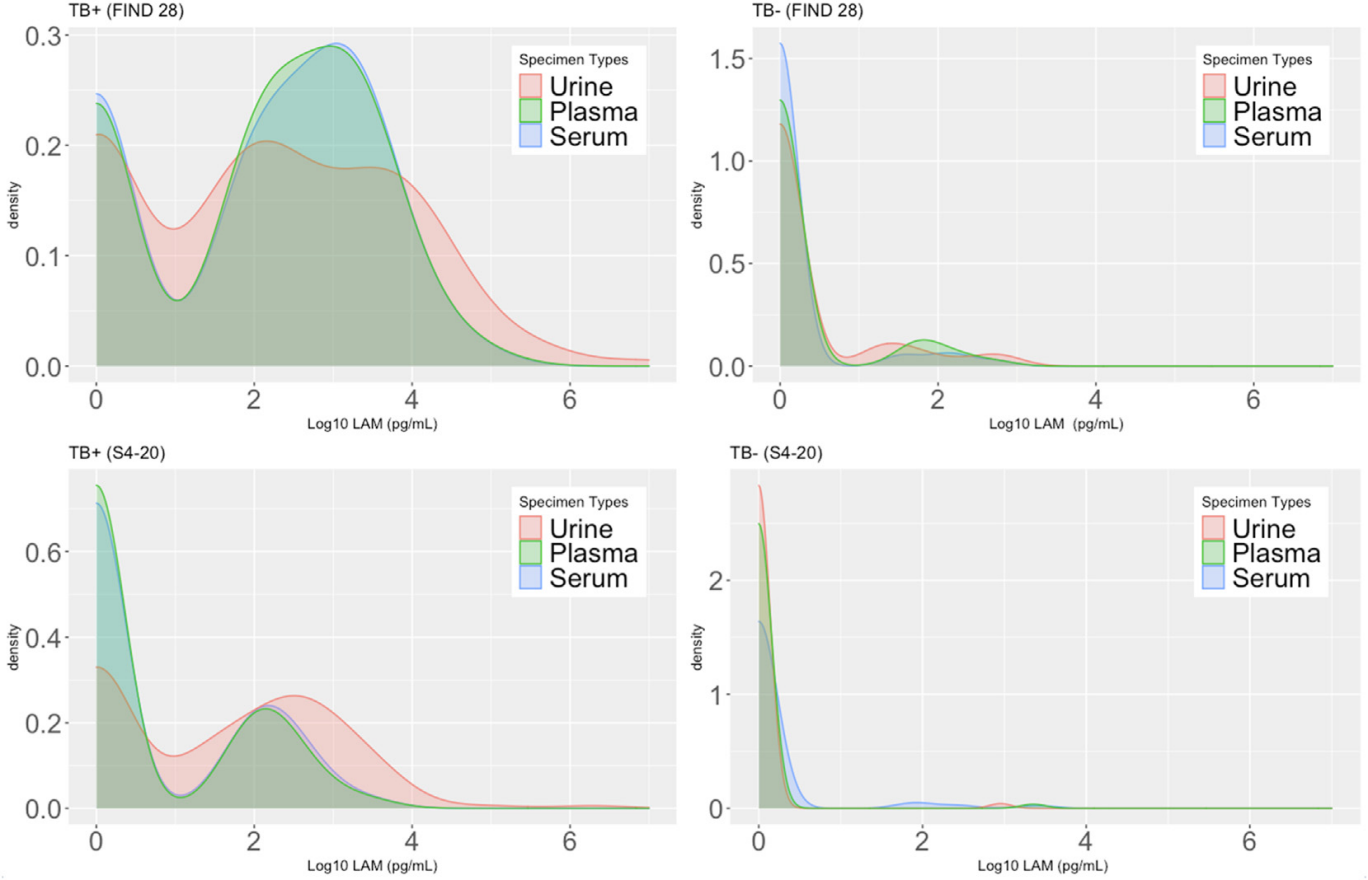
Density plots of LAM concentration (Log_10_ Transformed) by capture antibodies, specimen type, and TB status.

For the FIND 28 antibody, median LAM concentrations in urine, plasma, and serum were 137, 169 and 185 pg/mL, respectively, among those with microbiologically-confirmed TB (Table 2). For the S4–20 antibody, median LAM concentrations in urine, plasma, and serum were 52, 0 and 0 pg/mL, respectively, among TB-positive participants. Density plots showed distribution of plasma and serum LAM concentrations were more similar to each other than to urine LAM (Fig. 2). There were also higher proportions of blood samples than urine samples among TB-positive participants with no LAM detected by S4–20, according to the higher peaks at zero in the density plot.

**Table 2:** LAM concentrations (pg/mL) in urine, plasma, and serum by capture antibody among TB-positive participants (N = 138).

	Mean	SD	Median	IQR	Min	Max
**FIND 28**						
All TB+ (N = 138)						
Urine	82,002	831,465	137	0–3222	0	9,760,414
Plasma	2610	10,574	169	0–1496	0	111,111
Serum	2417	9031	185	0–1496	0	91,871
TB+ and HIV+ with CD4 <200 cells/mm^3^ (N = 67)						
Urine	168,105	1,191,804	2939	222–2939	0	9,760,414
Plasma	4983	14,813	1327	194–3089	0	111,111
Serum	4558	12,581	1489	197–2907	0	91,871
TB+ and HIV+ with CD4 ≥200 cells/mm^3^ (N = 30)						
Urine	58	101	12	12–87	0	460
Plasma	384	1665	0	0–56	0	9126
Serum	434	1935	0	0–76	0	10,639
TB+ and HIV-negative (N = 29)						
Urine	496	2410	0	0–55	0	13,012
Plasma	195	276	83	0–150	0	883
Serum	230	361	73	0–285	0	1305
**S4–20**						
All TB+ (N = 138)						
Urine	15,417	167,868	52	0–437	0	1,971,167
Plasma	116	413	0	0–73	0	3296
Serum	125	426	0	0–82	0	3821
TB+ and HIV+ with CD4 <200 cells/mm^3^ (N = 67)						
Urine	31,662	240,777	332	62–1065	0	1,971,167
Plasma	187	562	0	0–138	0	3296
Serum	191	564	0	0–129	0	3821
TB+ and HIV+ with CD4 ≥200 cells/mm^3^ (N = 30)						
Urine	20	70	0	0–0	0	370
Plasma	24	87	0	0–0	0	442
Serum	27	108	0	0–0	0	570
TB+ and HIV-negative (N = 29)						
Urine	64	121	0	0–61	0	485
Plasma	77	233	0	0–0	0	1202
Serum	111	313	0	0–116	0	1641

SD, Standard Deviation; IQR, Interquartile Range; Min, Minimum; Max, Maximum.

When stratifying TB-positive participants by HIV status and CD4 counts, PLHIV with CD4 <200 cells/mm^3^ had much higher median urine LAM concentrations detected by FIND 28 (2939 pg/mL) than PLHIV with CD4 ≥200 cells/mm^3^ (12 pg/mL) and participants without HIV (0 pg/mL). Similarly, for the S4–20 antibody, the median urine LAM concentration for PLHIV with CD4 <200 cells/mm^3^ was 332 pg/mL, whereas the median was 0 pg/mL among both PLHIV with CD4 ≥200 cells/mm^3^ and participants without HIV.

In summary, LAM concentrations were measurable in plasma and serum for some TB-positive participants, and results varied by capture antibodies (Fig. 2), HIV status and CD4+ T cell count (Table 2). Overall, median LAM concentrations were much lower in blood specimens (as compared to urine specimens) among PLHIV with CD4 <200 cells/mm^3^. Among both PLHIV with CD4 ≥200 cells/mm^3^ and participants without HIV, LAM concentrations in blood and urine were comparably low, regardless of capture antibody. LAM concentrations in urine, plasma and serum among 68 TB-negative participants are summarized in Table S3.

### Correlation of LAM concentrations across biospecimens

Correlation coefficients of LAM for each pair of specimens are summarized in Table S4. For LAM detected by FIND 28, there was high correlation (Spearman's r = 0.91 (95% CI: 0.90–0.97)) between plasma and serum. Urine LAM had similar correlations to plasma (r = 0.74 (95% CI: 0.64–0.79)) and serum (r = 0.75 (95% CI: 0.66–0.81)) LAM. As for S4–20, the Spearman's correlation coefficients were consistently lower than those for FIND 28, with r = 0.79 (95% CI: 0.66–0.87) between plasma and serum, r = 0.60 (95% CI: 0.47–0.70) between urine and plasma, and r = 0.56 (95% CI: 0.41–0.66) between urine and serum.

### Diagnostic accuracy of LAM in urine and blood

For both antibodies, the diagnostic sensitivity of urine LAM was much higher among PLHIV with CD4 <200 cells/mm^3^, as compared to either PLHIV with CD4 ≥200 cells/mm^3^ or participants without HIV (Table 3). However, we found specificities of urine LAM were high among PLHIV, regardless of CD4+ T-cell count, for FIND 28 (88% vs 86%) or for S4–20 (96% vs 100%). Since all 29 HIV-negative participants were TB-positive, we were not able to calculate the specificities among this subgroup. Of note, the sensitivity of urine LAM was comparable between PLHIV with CD4 ≥200 cells/mm^3^ (43%, 95% CI: 25%–63%) and participants without HIV (38%, 95% CI: 21%–58%) only for FIND 28. Urine LAM detected by S4–20 demonstrated significantly higher sensitivity among participants without HIV (41%, 95% CI: 24%–61%) than PLHIV with CD4 ≥200 cells/mm^3^ (20%, 95% CI: 8%–39%).

**Table 3:** Diagnostic accuracy of FIND 28 and S4–20 capture antibodies for LAM detection in urine, plasma, serum for classification of pulmonary TB disease.

	Urine (N = 210)		Plasma (N = 209)		Serum (N = 207)	
	n/N	% (95% CI)	n/N	% (95% CI)	n/N	% (95% CI)
**FIND 28**						
HIV+ & CD4 <200 cells/mm^3^						
Sensitivity	61/67	91 (82–97)	59/67	88 (78–95)	61/67	91 (82–97)
Specificity	22/25	88 (69–97)	24/27	88 (68–97)	19/24	79 (58–93)
HIV+ & CD4 ≥200 cells/mm^3^						
Sensitivity	13/30	43 (25–63)	7/30	23 (10–42)	12/30	40 (23–59)
Specificity	38/44	86 (73–95)	42/44	95 (85–99)	42/43	98 (88–100)
HIV-negative						
Sensitivity	11/29	38 (21–58)	16/29	55 (36–74)	17/29	59 (39–76)
Specificity	0/0	NA	0/0	NA	0/0	NA
**S4–20**						
HIV+ & CD4 <200 cells/mm^3^						
Sensitivity	59/67	88 (78–95)	29/67	43 (31–56)	31/67	46 (34–59)
Specificity	24/25	96 (80–100)	23/24	96 (79–100)	21/24	88 (68–97)
HIV+ & CD4 ≥200 cells/mm^3^						
Sensitivity	6/30	20 (8–39)	3/30	10 (2–27)	3/30	10 (2–27)
Specificity	44/44	100 (92–100)	44/44	100 (92–100)	42/43	98 (88–100)
HIV-negative						
Sensitivity	12/29	41 (24–61)	7/29	24 (10–44)	9/29	31 (15–51)
Specificity	0/0	NA	0/0	NA	0/0	NA

CI, Confidence Interval; NA, Not Applicable.

For FIND 28, plasma and serum LAM showed similar sensitivities and specificities to urine LAM only among PLHIV with low CD4 counts. For S4–20, much lower sensitivities from S4–20 were observed for plasma and serum LAM (43% and 46%) than urine LAM (88%). We also found relatively higher sensitivities among HIV-negative participants than PLHIV with CD4 ≥200 cells/mm^3^ for LAM detected in plasma and serum by both capture antibodies, suggesting potential differences of LAM distribution between these two groups.

### LAM benchmarks for optimal diagnostic accuracy

In our overall tested population, the diagnostic performance of urine LAM by FIND28 was similar to that of plasma and serum LAM for all selected participants (Table 4). For FIND 28, we estimated optimal thresholds for diagnostic accuracy of LAM for TB classification at 15.48, 39.31 and 40.64 pg/mL in urine, plasma and serum. respectively. However, adding additional LAM thresholds to the lab-detection thresholds only slightly improved the sensitivities in all three specimens, and had minimal impact on specificities. The best diagnostic accuracy for the FIND 28 antibody was testing serum specimens, which had a sensitivity of 70% and specificity of 94% with an AUC at 0.82 (95% CI: 78%–87%) (Figure S2).

**Table 4:** Diagnostic accuracy of LAM by FIND-28 and S4–20 (Lab detection thresholds vs Additional ROC curve optimal cutoff).

	Urine (N = 210)	Plasma (N = 209)	Serum (N = 207)
	% (95% CI)	% (95% CI)	% (95% CI)
**FIND 28**			
**Lab detection thresholds**			
Sensitivity	67 (59–72%)	65 (57–73%)	70 (61–77%)
Specificity	86 (76–93%)	93 (84–98%)	91 (82–97%)
**Additional ROC optimal cutoff point**	15.48	39.31	40.64
Sensitivity	70 (62–77%)	70 (62–78%)	70 (62–78%)
Specificity	85 (76–93%)	90 (82–97%)	94 (88–99%)
**S4–20**			
**Lab detection thresholds**			
Sensitivity	62 (53–70%)	30 (22–38%)	33 (26–42%)
Specificity	99 (93–100%)	99 (92–100%)	94 (86–98%)
**Additional ROC optimal cutoff point**	4.48	21.22	16.60
Sensitivity	62 (53–70%)	31 (24–39%)	33 (25–41%)
Specificity	99 (96–100%)	99 (96–100%)	94 (88–99%)

ROC, Receiver Operating Characteristic; CI, Confidence Interval.

For the S4–20 antibody, we observed consistently high specificities at 99% (95% CI: 93%–100%), 99% (95% CI: 92%–100%), and 94% (95% CI: 86%–98%) in urine, plasma, and serum, respectively. However, the sensitivity of LAM in urine (62%, 95% CI: 53%–70%) appeared to be higher than that for plasma (30%, 95% CI: 22%–38%) and serum (33%, 95% CI: 26%–42%). Additional LAM thresholds by S4–20 (4.48 pg/mL for urine, 21.22 pg/mL for plasma, and 16.60 pg/mL for serum) had no improvement on diagnostic accuracy over lab-based detection thresholds. The best diagnostic accuracy by the S4–20 antibody was for urine specimens, which had an AUC at 0.80 (95% CI: 75%–84%).

### Venn diagrams and parallel testing

Overall, 35 (25.4%) of the 138 TB-positive participants had no LAM detected in urine, either by FIND 28 or S4–20 (Figures S3–S5). In addition, none of those 35 participants were urine positive by the Determine™ TB LAM Ag assay (Table S5). This subgroup was also disproportionately PLHIV with CD4 counts above 200 cells/mm^3^ (72%). The percentages of participants with no LAM detected by either the FIND 28 or S4–20 antibody were similar for plasma (33.3%) and serum (29.7%). Compared with testing with only one capture antibody, parallel testing of LAM by both FIND 28 and S4–20 capture antibodies led to a higher sensitivity in urine (75%) no improvement in sensitivity for plasma (67%) and serum (70%) specimens, and no improvement in specificity (Table S6).

## Discussion

This study has demonstrated the detection of LAM in three time-matched non-sputum biospecimens from a real-world clinical cohort using a high sensitivity ECL immunoassay for diagnosing pulmonary TB disease. Measured LAM concentrations were significantly varied between two leading capture antibodies (FIND 28 and S4–20), by urine and blood specimen types, as well as among PLHIV with and without immunodeficiency and HIV-negative adults. While LAM concentrations were generally higher among PLHIV with immunosuppression, as compared to PLHIV with CD4 ≥200 cells/mm^3^ or participants without HIV, the concentration of LAM among people with microbiologically-confirmed pulmonary TB disease was low. Overall, these results provide evidence of low level detection of LAM in three specimen types, among people living with and without HIV, and offer benchmarks for the development of new rapid LAM diagnostic tests. However, for a rapid LAM test to be a useful diagnostic biomarker of pulmonary TB disease, prudent antibody selection by biospecimen type and pre-analytical LAM concentration or signal amplification will be necessary to achieve diagnostic accuracy targets.

Since sputum can be difficult to obtain for pauci-bacillary disease and in young children,^5^, ^6^, ^7^^,^^21^ the WHO has called for accurate, non-sputum TB biomarker tests to facilitate same-day treatment initiation. In this cohort, detecting LAM by S4–20 in urine, or by FIND 28 in plasma/serum nearly achieved diagnostic accuracy criteria to meet the WHO's Target Product Profiles (TPP) for rapid biomarker-based non-sputum tests (>65% sensitivity, >98% specificity).^22^^,^^23^ However, the minimum criteria set in the WHO's TPP focus on test accuracy, and do not account for ability to provide a sample or diagnostic yield. Rapid diagnostic tests that have lower accuracy, but are easily obtainable, could improve diagnostic yield and impact TB case detection rates.^24^^,^^25^ One study has shown urine LAM testing to have a higher diagnostic yield than sputum Xpert testing.^3^ Therefore, rapid tests with lower diagnostic accuracy may warrant clinical studies that can measure the individual and public health impact of non-sputum TB diagnostics.

Few other studies have evaluated the diagnostic performance of non-sputum LAM detected by newly identified LAM antibodies using the ECL assay.^19^^,^^20^^,^^26^ In one retrospective study using 81 biobanked urine samples collected from four countries, the FIND 28 antibody showed 75% sensitivity and 66% specificity, while the S4–20 antibody had 93% sensitivity and 97% specificity.^18^^,^^19^ These reported sensitivities were slightly higher than the results reported in our study for both FIND 28 (67%) and S4–20 (62%). The spectrum of disease included in the prior study may have contributed to the higher sensitivity observed, since microbiologically-confirmed TB positive was defined as positive on both smear-microscopy and at least one culture. The definition of microbiologically confirmed TB in the present study better aligns with a real-world diagnostic population. Among HIV-negative outpatients from South Africa and Peru, another study of the S4–20 antibody reported similar diagnostic accuracy to our estimates for urine LAM [sensitivity 66.7% (95% CI: 57.5%–74.7%); specificity 98.1% (95% CI: 95.6%–99.2%)].^27^ A study in Vietnam showed low sensitivity (39%, 95% CI: 33%–44%) among HIV-negative adults.^28^ Despite the observed between-study variability in diagnostic sensitivity, these studies consistently demonstrate LAM to be found in low levels in urine, when using a ECL assay with a LOD threshold of 5–10 pg/mL. Motivated by the detection of elevated levels of LAM in exhaled breath condensate samples from individuals diagnosed with TB, ongoing research efforts are directed toward creating a TB test utilizing exhaled breath analysis.^29^

In our study, we established relatively low benchmark LAM targets (4–40 pg/mL) for optimal LAM detection in urine or blood specimens. However, there remained a significant percentage (20–30%) of participants with MRS-confirmed pulmonary TB disease who had no LAM detected by either capture antibody across all three specimen types. Most of these people were PLHIV with CD4 ≥200 cells/mm^3^ or participants without HIV, who comprise the majority of TB cases worldwide. We also observed lower LAM concentrations in blood for PLHIV with CD4 ≥200 cells/mm^3^, as compared to participants without HIV. This finding contradicts the expected immunological advantage of the latter group in terms of LAM-binding antibody production, further research may still be needed to explain the poor diagnostic accuracy of LAM among PLHIV with CD4 ≥200 cells/mm^3^.^30^ Differences in LAM concentration between participants without HIV and PLHIV with CD4 ≥200 cells/mm^3^ may be due to the pauci-bacillary nature of TB among PLHIV or changes in immunological function among people who have re-established their CD4 T-cell population.

Our study also demonstrated differential immunoreactivity between LAM capture antibodies across various biospecimens. Notably, FIND 28 detected similar levels (medians) of LAM with consistent sensitivities across all three investigated biospecimens. In contrast, S4–20 displayed significantly lower sensitivities with less LAM detected in blood compared to urine. A similar pattern was observed in the prior study of biobanked urine and serum samples for S4–20.^18^ The observed different immunoreactivity to LAM between FIND 28 and S4–20 may be related to different LAM epitopes targeted by each antibody.^19^^,^^20^ This emphasizes the need for careful consideration of antibody and biospecimen selection when developing TB diagnostics, and future studies may focus more on epitope mapping for selecting optimal combinations of antibodies for LAM detection.

A potential limitation of our study is that we only used the MRS instead of a composite reference standard to define TB positivity. This may have led to underestimation of specificities because some clinical TB cases with detectable LAM levels would be misclassified as TB-negative. Nevertheless, with molecular testing (Xpert MTB/RIF Ultra) and *Mtb* culture available to all our participants, the impact on specificities should be small since few participants would have been diagnosed with TB disease without having microbiological evidence. All sputum-negative participants were also followed for 3 months to ensure they did not meet a clinical definition of having TB disease. Since all TB-negative participants were PLHIV, we were unable to assess the specificities of LAM among HIV-negative populations. Furthermore, LAM concentrations may be different among people who have TB disease, but are unable to produce expectorated sputum specimens for TB molecular testing and culture.

In conclusion, this study has demonstrated that detection of LAM is achievable in non-sputum specimens in the setting of pulmonary TB among most people living with and without HIV. However, the detectable LAM concentrations are relatively low and depend on the antibody, biospecimens, and patient characteristics. For a next-gen LAM assay to be useful for TB diagnosis, the assay may need to include either analyte concentration or signal amplification to achieve the desired diagnostic accuracy targets. Additionally, mapping target epitopes of the LAM glycolipid for widespread screening and selecting newly identified antibodies of the most conserved regions may be useful. Regardless, the real-world performance of future LAM assay should not only measure clinical accuracy, but also evaluate diagnostic yield and population health outcomes.

## Contributors

PKD and DW acquired study funding. PKD, AES and DW designed the study. ZPM, ZN, MWN, NCN, JC, CA, RB and BG conducted the study and collected data. AP provided the detector antibody. XN, EB and BG curated the data. XN, KKT designed the statistical analyses and XN did the formal analysis. JFM was responsible for project administration. PKD, RD and DW were responsible for supervision and validation. XN prepared the tables and figures. XN, RD and PKD wrote the original draft of the manuscript. XN and EB accessed and verified the data. All authors had full access to all the data in the study and had read and approved the final version of the manuscript.

## Data sharing statement

The study data consist of individual participant data accompanied by a comprehensive data dictionary defining each field within the dataset. De-identified versions of this data can be made available upon reasonable request. However, accessing the data will require approval of a formal proposal submitted to the study team, followed by the signing of a data access or sharing agreement.

## Declaration of interests

PKD and DW declare receiving grant funding, paid to the institution, from the Bill and Melinda Gates Foundation. PKD reports receiving consulting fees from ThermoFischer, InBios Internation, and Abbott Diagnostics, and advisory board fees from Abbvie and Cepheid. Rutgers University (AP) has a patent on the use of the A194-01 antibody for diagnosis of TB infections.
