# Supplementary material for: Real-world diagnostic accuracy of lipoarabinomannan in three non-sputum biospecimens for pulmonary tuberculosis disease
Source: eBioMedicine. 2024 Sep 26;108:105353. doi: 10.1016/j.ebiom.2024.105353 (PMC11481603; doi:10.1016/j.ebiom.2024.105353)

**Supplementary Materials**

**Table S1.** The estimated Limit of Blank (LOB) by antibody and specimen type.

|  | Urine | Plasma | Serum |
| --- | --- | --- | --- |
| FIND28 | 1.2 | 1.22 | 1.09 |
| S4-20 | 0.962 | 1.11 | 0.98 |

**Table S2.** The Limit of Detection (LOD) for ECL plates used for Urine, Plasma, and Serum LAM testing.

| **Urine** | | | |  | **Plasma** | | | |  | **Serum** | | | |
| --- | --- | --- | --- | --- | --- | --- | --- | --- | --- | --- | --- | --- | --- |
| ***FIND-28*** | | ***S4-20*** | |  | ***FIND-28*** | | ***S4-20*** | |  | ***FIND-28*** | | ***S4-20*** | |
| N | LOD | N | LOD |  | N | LOD | N | LOD |  | N | LOD | N | LOD |
| 20 | 3.27 | 14 | 3.34 |  | 13 | 10.52 | 12 | 14.08 |  | 8 | 20.28 | 1 | 8.08 |
| 16 | 5.50 | 12 | 6.12 |  | 13 | 19.40 | 13 | 21.31 |  | 16 | 24.31 | 14 | 22.79 |
| 14 | 6.71 | 14 | 7.18 |  | 8 | 22.77 | 12 | 25.72 |  | 15 | 25.35 | 9 | 22.88 |
| 12 | 8.39 | 13 | 7.32 |  | 15 | 27.54 | 8 | 32.53 |  | 16 | 31.87 | 15 | 25.29 |
| 13 | 8.70 | 10 | 9.16 |  | 15 | 28.96 | 15 | 36.06 |  | 14 | 32.33 | 12 | 31.77 |
| 10 | 8.73 | 3 | 9.39 |  | 17 | 30.94 | 17 | 37.92 |  | 9 | 35.98 | 8 | 37.37 |
| 19 | 9.53 | 13 | 9.77 |  | 12 | 35.36 | 15 | 40.04 |  | 18 | 39.74 | 2 | 38.76 |
| 14 | 11.86 | 1 | 12.87 |  | 11 | 37.86 | 19 | 40.65 |  | 12 | 40.18 | 13 | 47.17 |
| 13 | 13.20 | 20 | 13.4 |  | 13 | 38.41 | 17 | 42.35 |  | 18 | 42.66 | 15 | 52.46 |
| 14 | 14.70 | 14 | 14.94 |  | 12 | 38.88 | 17 | 48.14 |  | 13 | 43.96 | 18 | 60.69 |
| 3 | 14.93 | 19 | 19.92 |  | 17 | 41.22 | 17 | 49.28 |  | 1 | 53.74 | 18 | 70.41 |
| 16 | 22.18 | 16 | 20.89 |  | 10 | 43.37 | 13 | 57.48 |  | 17 | 55.26 | 16 | 78.92 |
| 12 | 26.08 | 20 | 21.79 |  | 17 | 51.86 | 13 | 70.79 |  | 15 | 56.46 | 9 | 83.57 |
| 20 | 27.06 | 16 | 23.53 |  | 17 | 61.33 | 10 | 110.42 |  | 9 | 57.2 | 16 | 86.66 |
| 1 | 31.43 | 12 | 23.82 |  | 19 | 91.58 | 11 | 112.64 |  | 16 | 64.24 | 16 | 91.97 |
| 13 | 31.94 | 13 | 24.92 |  |  |  |  |  |  | 8 | 88.23 | 8 | 104.16 |
|  |  |  |  |  |  |  |  |  |  | 2 | 95.47 | 17 | 111.98 |

**Table S3.** Average LAM concentrations (pg/mL) in urine, plasma, and serum by capture antibody among TB-negative participants (N=68).

|  | Mean | SD^*^ | Median | IQR | Min^*^ | Max^*^ |
| --- | --- | --- | --- | --- | --- | --- |
| **FIND 28** |  |  |  |  |  |  |
| Urine | 30 | 121 | 0 | 0-0 | 0 | 684 |
| Plasma | 19 | 74 | 0 | 0-0 | 0 | 543 |
| Serum | 16 | 74 | 0 | 0-0 | 0 | 560 |
| **S4-20** |  |  |  |  |  |  |
| Urine | 13 | 109 | 0 | 0-0 | 0 | 898 |
| Plasma | 33 | 273 | 0 | 0-0 | 0 | 2,253 |
| Serum | 43 | 305 | 0 | 0-0 | 0 | 2,508 |

^*^SD: Standard Deviation; IQR: Interquartile Range; Min: Minimum; Max: Maximum

**Table S4.** Correlation coefficients between urine, plasma and serum LAM by capture antibody (N=206).

|  | **Plasma & Serum** | |  | **Urine & Plasma** | |  | **Urine & Serum** | |
| --- | --- | --- | --- | --- | --- | --- | --- | --- |
|  | r | 95%CI |  | r | 95%CI |  | r | 95%CI |
| **FIND 28** |  |  |  |  |  |  |  |  |
| Spearman | 0.91 | 0.90-0.97 |  | 0.74 | 0.64-0.79 |  | 0.75 | 0.66-0.81 |
| Pearson | 0.99 | 0.98-0.99 |  | 0.15 | 0.01-0.28 |  | 0.19 | 0.05-0.32 |
| **S4-20** |  |  |  |  |  |  |  |  |
| Spearman | 0.79 | 0.66-0.87 |  | 0.60 | 0.47-0.70 |  | 0.56 | 0.41-0.66 |
| Pearson | 0.97 | 0.97-0.98 |  | 0.63 | 0.54-0.70 |  | 0.69 | 0.61-0.75 |

CI: Confidence Interval.

**Table S5**. Characteristics of TB participants vs TB participants with no urine LAM detected by FIND 28 & S4-20

|  | **TB-Positive**  **N=138**  **100%** | **TB-Positive (no urine LAM)**  **N=35**  **25%** |
| --- | --- | --- |
|  | **N (%)** | **N (%)** |
| Demographic |  |  |
| Age, mean (± SD) in years | 40 (± 14) | 45 (± 15) |
| Female sex | 57 (41%) | 15 (43%) |
| Clinical |  |  |
| Prior TB infection | 51 (37%) | 12 (34%) |
| Currently smoke tobacco | 19 (14%) | 4 (11%) |
| TB related symptoms |  |  |
| *Cough* | 116 (84%) | 29 (83%) |
| *Fever* | 98 (71%) | 26 (74%) |
| *Night sweats* | 97 (70%) | 22 (63%) |
| *Weight loss* | 123 (89%) | 31(89%) |
| *Having any TB-related symptom* | 134 (97%) | 34 (97%) |
| Recruitment location |  |  |
| Inpatient ward | 83 (60%) | 16 (46%) |
| Outpatient clinic | 55 (40%) | 19 (54%) |
| HIV status |  |  |
| HIV-infected | 109 (79%) | 22 (63%) |
| HIV-infected with CD4 <200 cells/mm^3^ | 67 (62%) | 3 (14%) |
| HIV-infected with CD4 ≥200 cells/mm^3^ | 30 (27%) | 16 (72%) |
| HIV-infected with CD4 missing | 12 (11%) | 3 (14%) |
| Tuberculosis Testing |  |  |
| Urine Determine TB LAM positive | 46 (33%) | 0 (0%) |
| Sputum smear microscopy positive^*^ | 9 (21%) | 1 (11%) |
| TB Microbiological Reference Standard |  |  |
| Sputum Xpert Ultra positive | 131 (95%) | 25 (71%) |
| Sputum *Mtb* culture positive | 81 (59%) | 15 (43%) |

**Table S6.** Diagnostic accuracy of parallel urine LAM testing by FIND-28 and S4-20

| **Parallel Testing** |  | **Urine** |  |  | **Plasma** |  |  | **Serum** |
| --- | --- | --- | --- | --- | --- | --- | --- | --- |
|  | n/N | **% (95% CI)** |  | n/N | **% (95% CI)** |  | n/N | **% (95% CI)** |
| **Positive: LAM detected by either FIND 28 or S4-20;**  **Negative: LAM NOT detected by both FIND 28 or S4-20** | | | | | | | | |
| Sensitivity | 103/138 | 75 (67-82) |  | 92/138 | 67 (58-74) |  | 97/138 | 70 (62-78) |
| Specificity | 60/68 | 88 (78-95) |  | 63/68 | 93 (84-98) |  | 62/68 | 91 (82-97) |

**Figure S1.** Violin Plots of LAM concentration (Log_10_ Transformed) by capture antibodies, specimen type, and TB status.


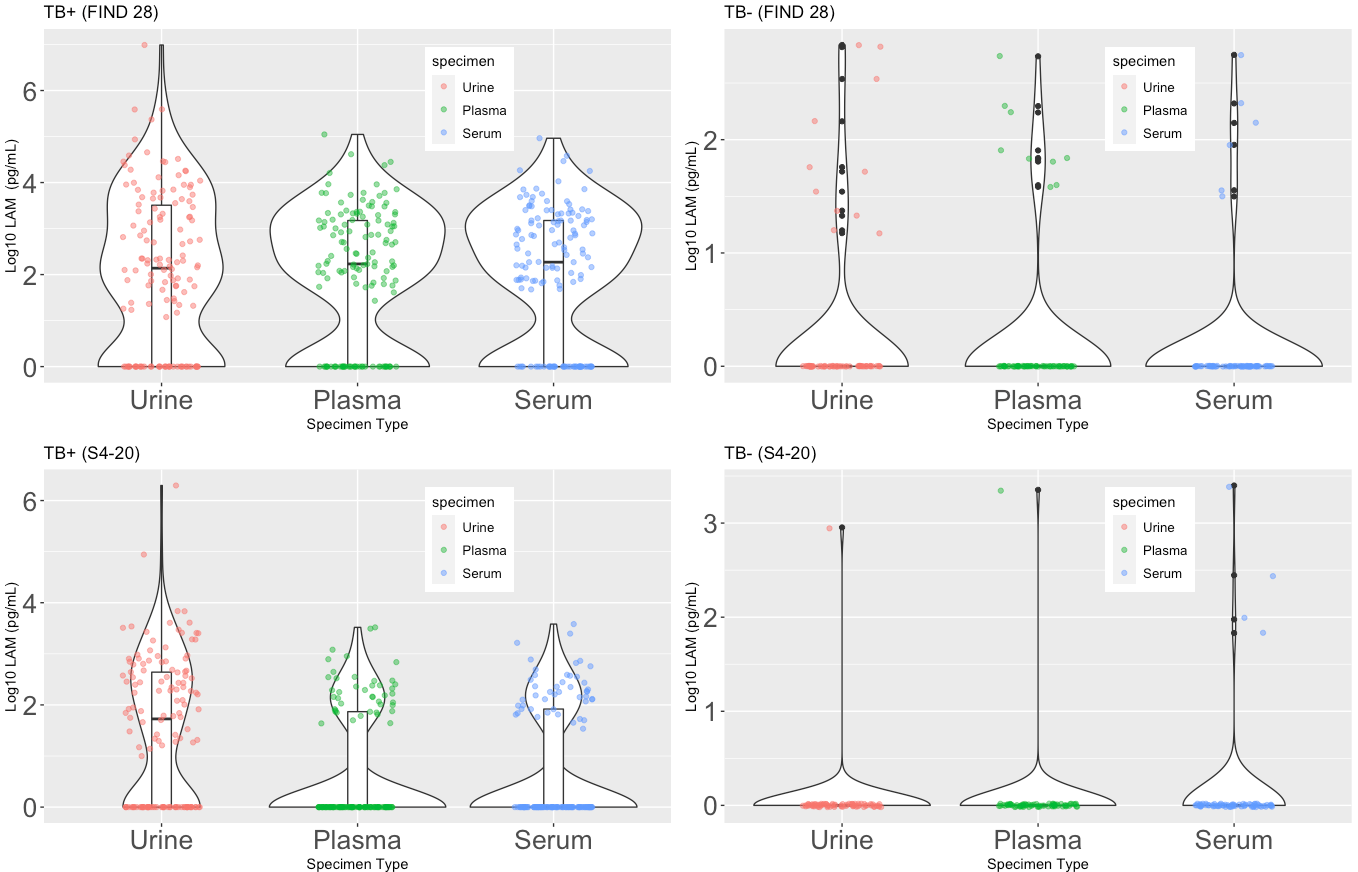


**Figure S2.** Receiver Operating Characteristic (ROC) Curves of LAM by capture antibodies, specimen type^*^.


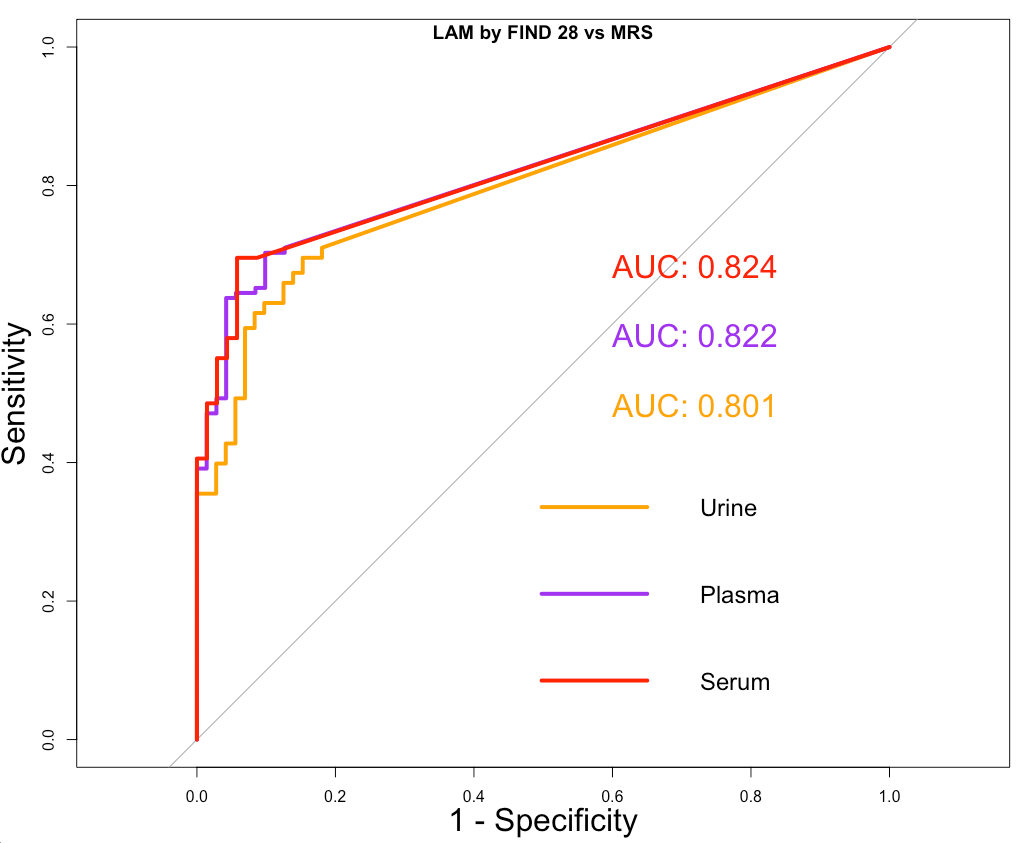

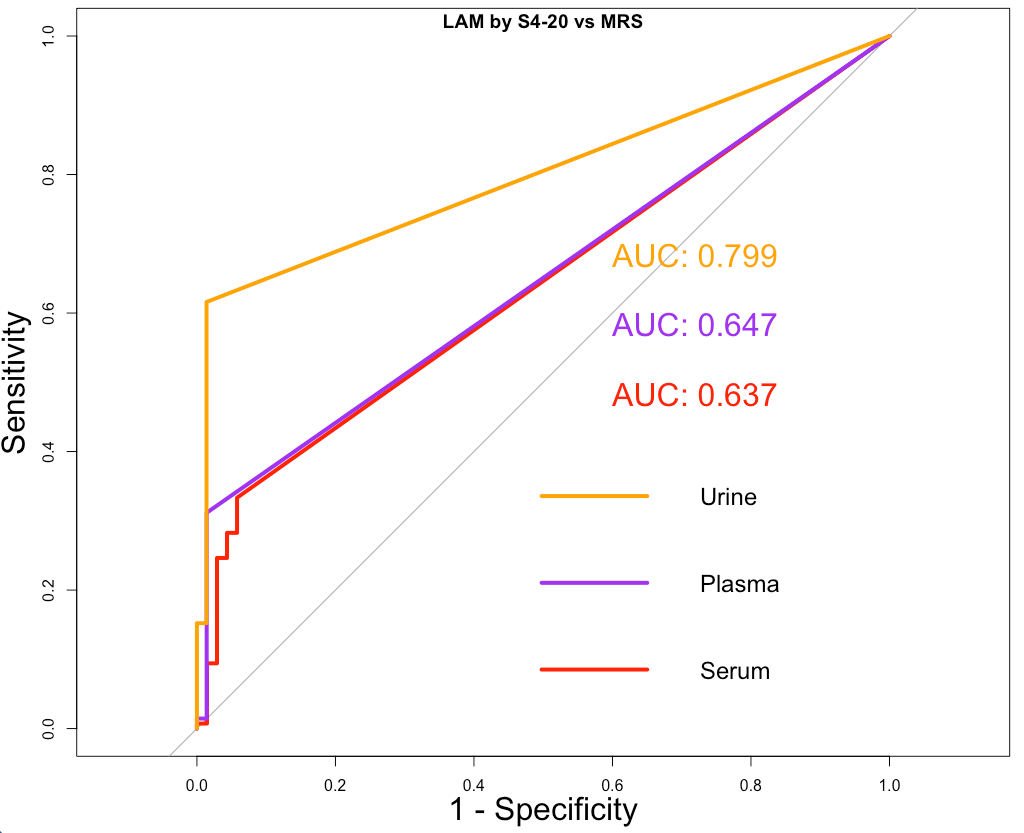


^*^AUC: Area Under the Curve.

**Figure S3.** Venn diagrams of LAM by capture antibodies, specimen type and TB status^*^.

***
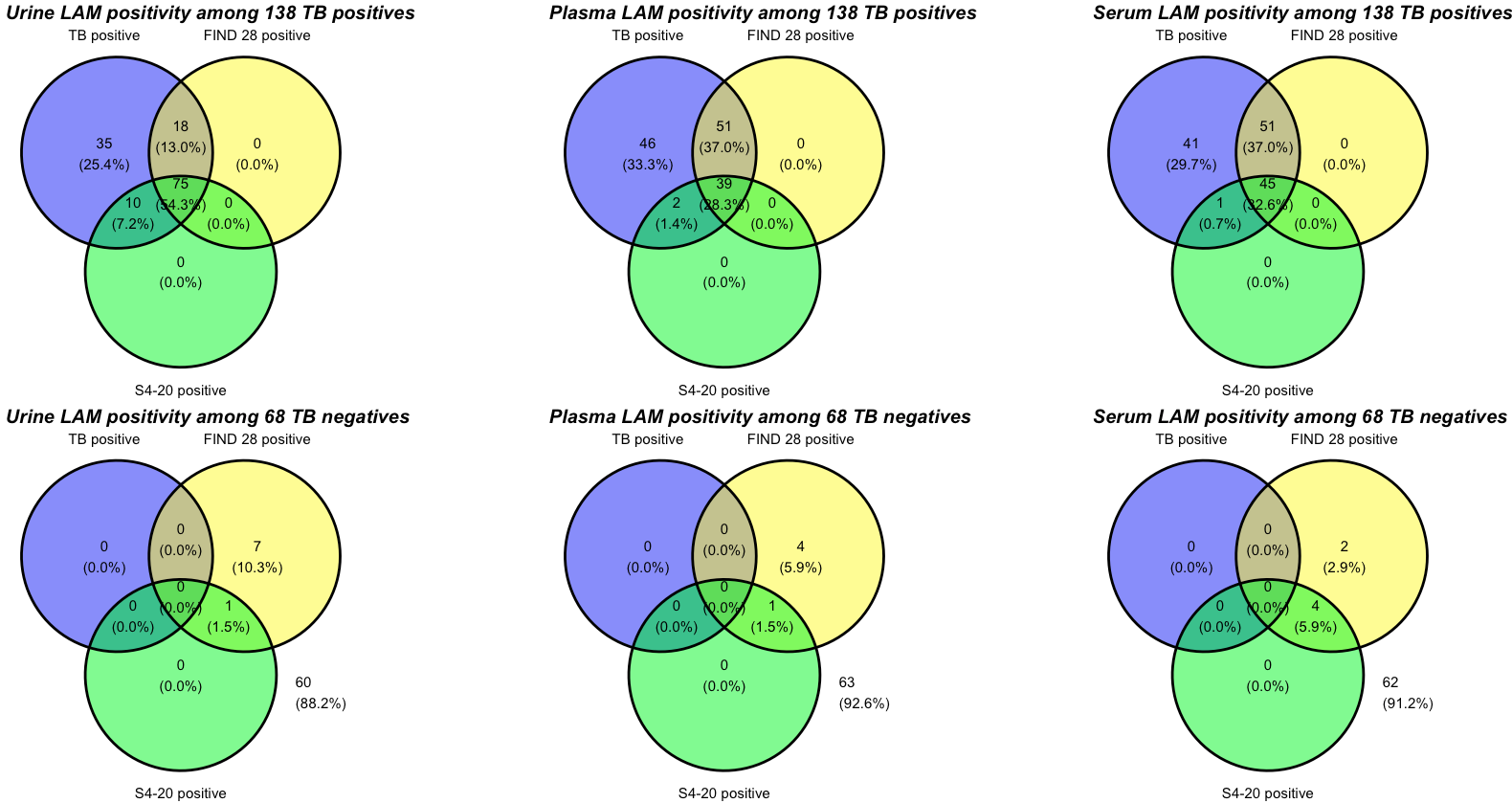
***

**Figure S4**. Venn diagram of TB positivity by MRS & LAM by FIND 28 in urine, plasma and serum.


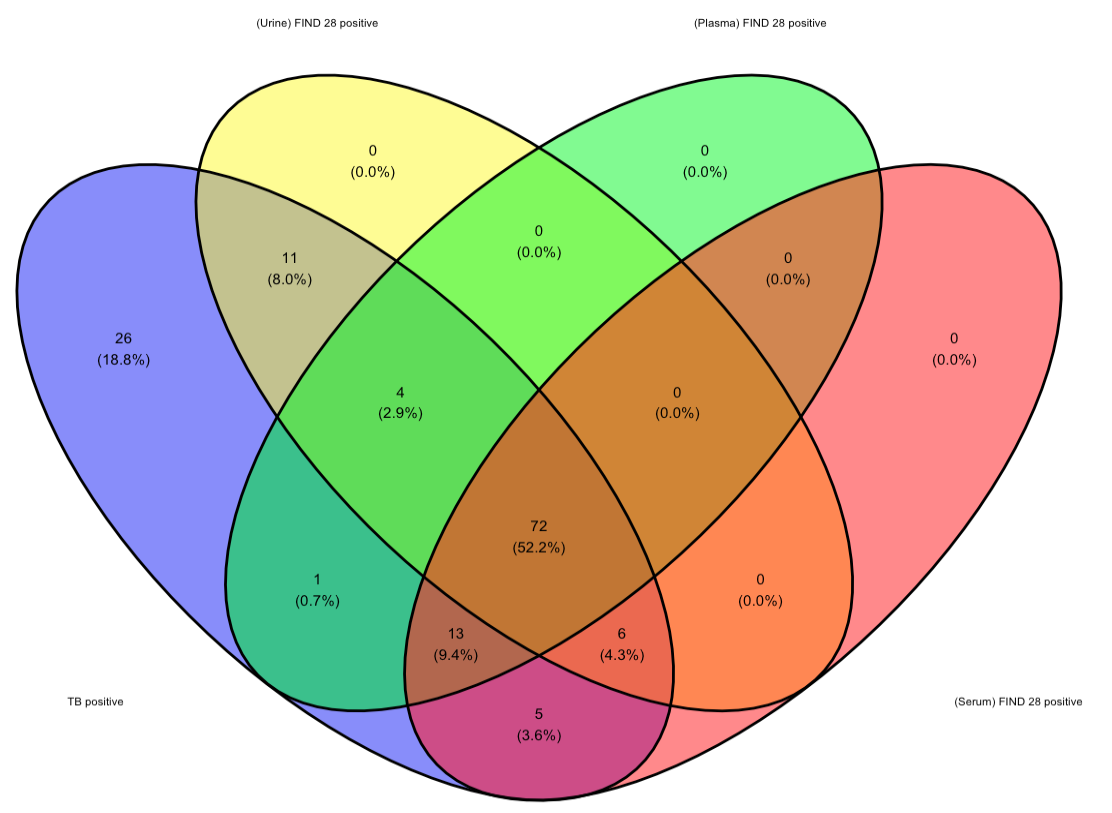


**Figure S5** Venn diagram of TB positivity by MRS & LAM by S4-20 in urine, plasma and serum.


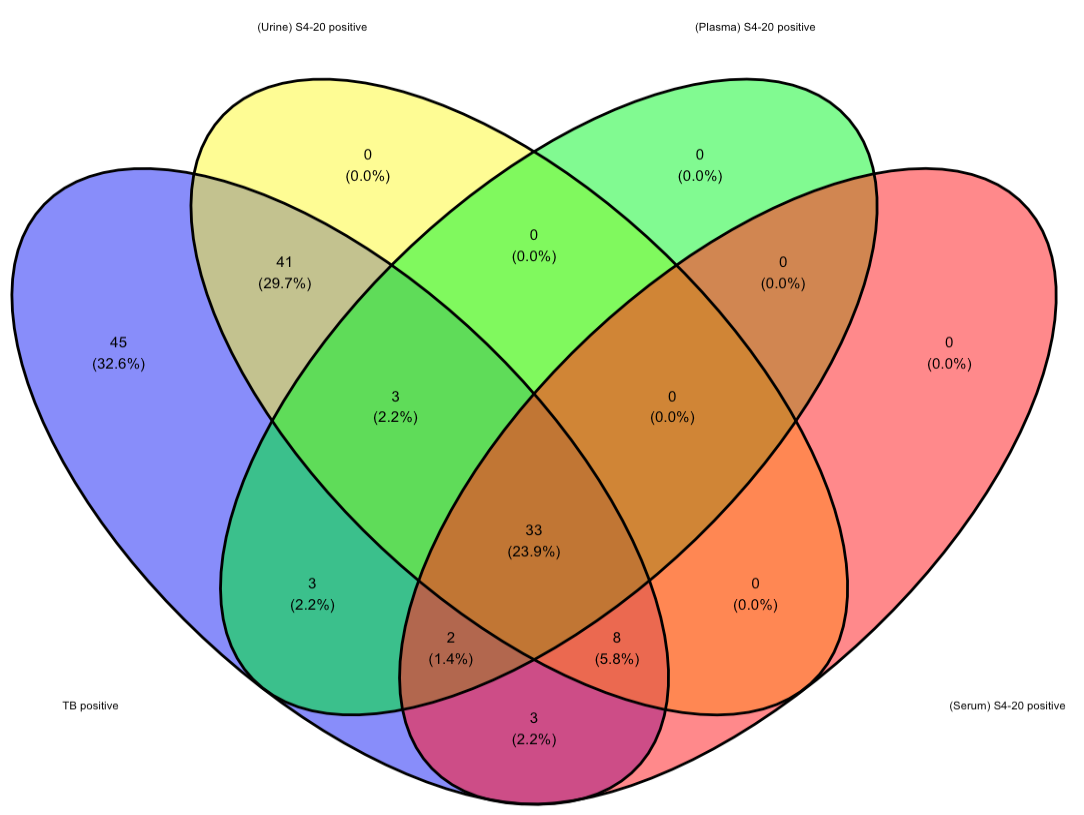

Supplement: Supplementary Files [file mmc1.docx]
